# A Disconnect between the Neurospirochetoses in Humans and Rodent Models of Disease

**DOI:** 10.1371/journal.ppat.1003288

**Published:** 2013-04-11

**Authors:** Juan C. Garcia-Monco, Jorge L. Benach

**Affiliations:** 1 Department of Molecular Genetics and Microbiology, Center for Infectious Diseases, Stony Brook University, Stony Brook, New York, United States of America; 2 Hospital de Galdacano, Vizcaya, Spain; Duke University Medical Center, United States of America

The human spirochetal diseases share many clinical similarities despite their diverse epidemiology. Syphilis is a sexually transmitted infection, whereas the leptospiroses and the borrelioses are zoonoses. The clinical similarities among the spirochetoses include a relapsing-remitting course of infection. The recurrent pattern of illness has three invariant features that are shared by all human spirochetoses. Entry of spirochetes is achieved through breaks in the skin or through mucous membranes. Cutaneous lesions such as the syphilitic chancre or the erythema migrans of Lyme disease constitute the earliest manifestations of these two diseases. In all three spirochetoses, there is dissemination to distant organs through tissues, blood, and other fluids. Late disease, often with intervening relapses and latent periods, can involve one or more organ systems. The spirochetal diseases also share tropisms for skin, nervous system, and heart and arteries.

The neurotropism of spirochetes is evident in syphilis, the human borrelioses (Lyme disease and relapsing fevers), and in leptospirosis. Spirochetes enter the central nervous system (CNS) very quickly. Their ability to cause neurological disease may be dependent on the virulence of the infecting strains and on the development of an inflammatory response. Yet, most of the current animal models for the spirochetoses either do not recreate the manifestations of the neurological spectrum or require special manipulations to establish infection (Figure 1). Thus, a major challenge for experimental approaches to the human neurological manifestations of the spirochetoses has been an inability or difficulties in reproducing all or some aspects of the human disease in animal models.

**Figure 1:**
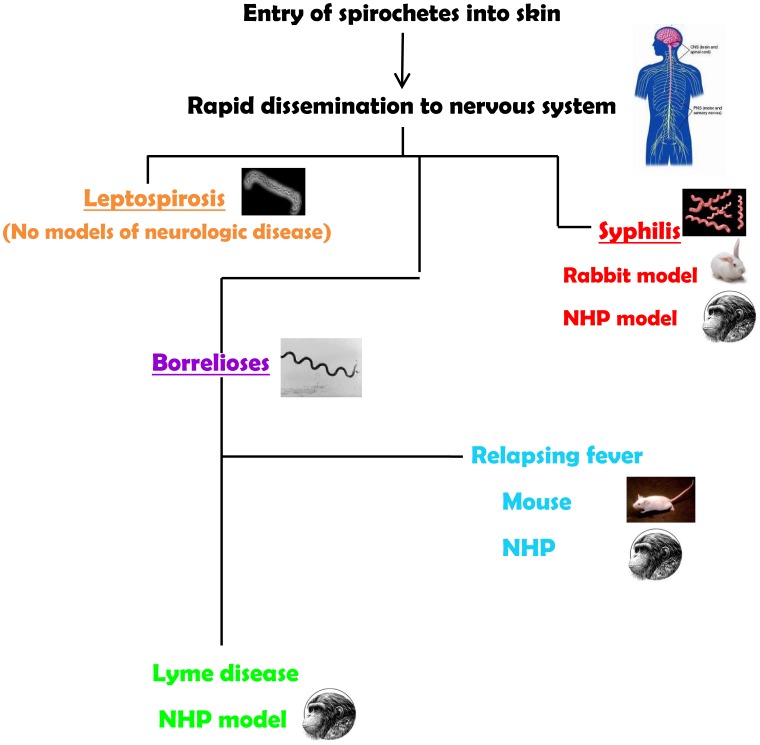
Summary of animal models for the neurospirochetoses.

## Leptospirosis

Leptospires enter the host through breaches in the skin as well as through mucous membranes [1]. The severity of leptospirosis is dependent on many factors including the infecting serovar and inoculum size. Clinical presentations in patients are variable, ranging from a self-limited infection to Weil's syndrome.

Weil's syndrome is a multi-organ disorder affecting the liver, lungs, and kidneys with marked vascular injury [2]. Leptospirosis presents with fever, chills, headache, nausea, vomiting, and myalgias. Enlarged liver, spleen, and lymph nodes are common signs. Eye involvement, specifically uveitis and optic neuritis, is common [3]. The second (biphasic) or immune stage of leptospirosis coincides with the development of antibodies against the organism. CNS involvement occurs during this phase and includes severe headache, encephalitis, facial palsy, and frequently, an aseptic meningitis [1]. Meningitis can be seen early after infection but is commonly observed after development of an antibody response [1].

The neurologic manifestations of leptospirosis may be associated with development of the antibodies. Given the availability of animal models, this phase of the disease could be potentially recreated; however, none of the laboratory animals used as models for leptospirosis develop neurologic manifestations either before or after formation of antibodies. Given that leptospires infect such a wide variety of animal species, it would have been expected that there would be excellent laboratory animal models. Hamsters and guinea pigs are highly susceptible and develop severe infection. Rats and mice, two of the main reservoirs of leptospires in nature, can only be infected in the laboratory with high doses of organisms. Dogs and nonhuman primates (NHP) can be infected as well. But, as mentioned, none of the laboratory animals develop obvious neurologic leptospirosis, despite the use of various routes of inoculation [4], [5]. This is a complex disparity for which there are no specific answers. Babudieri, in the 1958 study of animal reservoirs of leptospirosis, established a difference between the carrier and the true carrier conditions in which the former transmits (or sheds) leptospires following a brief infection, and the latter sheds for a longer period of time with or without a prior illness [6]. This may well mean that clinical neuroleptospirosis is rare among some reservoirs. In support of this possibility is the finding of a large number of apparently healthy urban rats that had both antibodies to and positive polymerase chain reactions (PCR) for leptospiral antigens in their brains (15 of 21 tested) in an area with human cases [7].

## Syphilis

While CNS invasion by *Treponema pallidum* occurs very early, neurosyphilis can take weeks or months to develop and may be asymptomatic, or include meningitis, cranial nerve involvement, and uveitis (usually near the time of secondary syphilis), or strokes, vertigo, and memory disturbances (late syphilis). Years later, chronic infection of the brain parenchyma or the posterior columns of the spinal cord may appear (tertiary neurosyphilis). In all cases, neurological manifestations are believed to be due to tissue damage resulting from inflammation and local immune response.

*T. pallidum* was recovered from the cerebrospinal fluid (CSF) of 30% of patients with early syphilis, indicating that invasion of the nervous system is a common and early event [8] and can include meningitis, visual and hearing manifestations, and facial palsy. Some patients with concurrent neurosyphilis and HIV infection have more severe manifestations of the neurosyphilis spectrum. This would indicate that immunity is important in controlling *T. pallidum* in the CNS or in modulating the severity of the disease, and this association could be exploited through the use of mouse models with specific immune deficiencies, and in NHP models using immune suppressants [9].

Two models have been developed for neurosyphilis. Both the rabbit and the NHP models use direct intrathecal inoculation, thereby bypassing the most likely manner of CNS entry through the blood brain barrier. Inoculation of rabbits via the intracisternal route resulted in CSF pleocytosis and in the presence of treponemes for several weeks. Likewise, uveitis has been reported in rabbits inoculated via the intradermal route [9]. Meningeal and ocular manifestations in this model recreate the early neurosyphilis in patients [10]. NHP were infected by the intrathecal route resulting in objective neurosyphilis findings that included pleocytosis in the CSF, persistence of organisms for several weeks, and changes in cytokine profiles. The rabbit and the NHP model are useful for studies on the responses to this organism within the CNS, although both models have the disadvantages of cost and ease of handling [11].

## The Borrelioses

The neurologic involvement of infection with *Borrelia burgdorferi* (Lyme neuroborreliosis) can also be divided into acute and chronic manifestations according to time of appearance relative to the onset of the disease [12]. The acute syndromes include cranial neuritis, meningitis, and radiculoneuritis [12]–[14]. Much less common, the chronic manifestations include a radiculoneuropathy (inflammatory disease of the spinal nerve roots), an encephalopathy characterized by impairment of cognitive functions and memory loss, and a leukoencephalitis [12], [15].

Both neurosyphilis and Lyme neuroborreliosis are characterized by an early invasion of the nervous system, as shown by experimental evidence in animal models [10], [16]. This is consistent with the finding of a clinically silent CSF pleocytosis in many patients at an early stage of infection, likely representing early nervous system invasion. Relapsing fever can also involve the nervous system, resulting in meningitis, facial nerve palsy, radiculitis, and encephalopathy, and is similar to the neurologic manifestations of Lyme disease [17], [18].

The murine model of Lyme disease does not reproduce any manifestations compatible with human neuroborreliosis. From its initial characterization, the mouse model of Lyme disease reproduced cutaneous infection (although not erythema migrans), carditis, and arthritis but not central or peripheral nervous system signs. This has been a major detriment to the study of neuroborreliosis in the murine setting, with its inherent advantages in genetics and immunological experimental approaches [19].

Relapsing fever (RF) is a multisystemic borrelioses that can occur in epidemic (louse-borne) and endemic (tick-borne) forms, and is caused by a variety of *Borrelia* species. A marked difference between relapsing fever and Lyme disease is the presence of recurrent spirochetemia in the former. The laboratory mouse has been used to reproduce the neuroborreliosis of relapsing fever experimentally for many years. The presence of large numbers of motile spirochetes in the circulation could be a factor in the breakdown of the blood brain barrier through waste products or mediators of inflammation so that penetration into the CNS could be achieved more easily. Immunocompetent and immunodeficient mice have been infected successfully with both old and new world species of relapsing-fever borrelia. Infection of mice with subsequent neurological manifestations can be achieved through cutaneous inoculation, the route that reproduces transmission by ticks [20]. The most common neurologic manifestation in mice is meningitis, with spirochetes detectable infrequently in the leptomeninges and in the cerebrospinal fluid [20], [21]. Despite the paucity of spirochetes in the brain, there is cerebral microgliosis that is more severe in immunodeficient mice. This finding emphasizes the role of the immune response in the development and severity of relapsing fever. Of note is the vestibular dysfunction of mice infected with relapsing-fever borrelia. While spirochetes have been observed in cranial and peripheral nerves [22], there are no studies documenting peripheral nerve disease in mice.

The protean nature of *B. burgdorferi* infection (Lyme disease) including the nervous system can be reproduced in rhesus monkeys (*Macaca mulatta*). Immunocompetent and immunosuppressed NHP can be infected via tick bite and by cutaneous injection of *B. burgdorferi*. The similarity between the signs of Lyme disease and those that have been described in the NHP model is a distinct advantage for studies on experimental pathogenesis. NHP exhibit early and disseminate manifestations that are characteristic of Lyme disease [23], [24]. Neuroborreliosis is recreated in this animal model, and *B. burgdorferi* disseminate to the brain, brainstem and cerebellum, spinal cord, and the meninges [25], [26]. Peripheral neuroborreliosis was documented through nerve conduction studies performed on NHP inoculated with *B. burgdorferi*. Conduction velocities were recorded on motor nerves of the extremities at various stages of the infection, and axonal multifocal neuropathies of the arms and legs were confirmed along with evidence of denervation [24]. *B. burgdorferi* was detected in tissues of the peripheral nervous system in normal and immunosuppressed animals [25]. The histopathologic and immunohistochemical features of early and late neuroborreliosis of the peripheral nervous system showed that neuritis involving multiple nerves was the most consistent manifestation with immune cell infiltration [27].

## What to Do about Improving Animal Models of Neurospirochetoses

There are common themes to the neurological manifestations of the spirochetoses. Acute and subacute meningitis, facial palsy, and peripheral neuropathic disease are the common denominators to neuroborreliosis, neurosyphilis, and leptospirosis. Improvements in experimental models for the neurospirochetoses would be very important to sort out some of the controversial manifestations of human Lyme disease that are associated with the nervous system.

Although at least two NHP models for neurosyphilis and neuroborreliosis and a murine model for relapsing fever recreate all or part of the human spectrum of nervous system manifestations, there are some constraints that preclude their widespread use. In the case of the NHP models, issues of cost and handling complexity are two of the main impediments for their frequent use. In the case of neurosyphilis, as has been pointed out earlier, the limiting factor is in the entry of *T. pallidum* into the nervous system. This is an area that could be investigated with new in-vitro systems to identify specific requirements for treponemal interactions with the brain vasculature.

In the case of the NHP models of neurological Lyme disease, it is of note that immunosuppression leads to greater severity and greater spirochetal burden. To obviate the complex NHP model systems, we propose revisiting murine models with single or multiple deletions of genes involved in the innate and acquired immune systems.

Where to begin the trial and error experiments that would be required to establish a permissive murine model of Lyme neuroborreliosis? Just as in the NHP models, two limiting factors in mice may be related to entry into the nervous system. Entry of other bacteria into the CNS has been studied in mice with molecular markers in the vasculature for which deletions exist. Likewise, experiments using direct inoculation of *B. burgdorferi* into the nervous system have not been pursued vigorously, except with NHP. Distinct signs of experimental CNS disease in mice that are less obvious than meningitis or encephalitis, such as flaccid tails or various degrees of limb paralysis, could be documented in mouse models, as was done in a recent study with *B. hermsii* [28]. For that matter, peripheral nervous system manifestations that may be more subtle or difficult to characterize need to be explored in mice as well. For Lyme neuroborreliosis, we know that neither a meningitis nor encephalitis has been documented in mice. There are a number of experimental approaches that could be used to document peripheral nerve and ocular disorders that have not been tried in the mouse model of Lyme borreliosis. Until all of these possibilities have been explored, it is difficult to admit that the neurological manifestations that are so prominent in the human spirochetoses cannot be recreated in murine models.
